# Microfluidic Chip for High-Throughput Microstructure Detection of Precursor Particles

**DOI:** 10.3390/mi17080932

**Published:** 2026-08-04

**Authors:** Fenglin Han, Jing Wang, Jinlong Wu, Jing Yang, Hu He, Zhi Chen

**Affiliations:** College of Mechanical and Electrical Engineering, Central South University, Changsha 410083, China; hanfl@csu.edu.cn (F.H.); wangjing7110@163.com (J.W.); wuj_long@163.com (J.W.); 243701030@csu.edu.cn (J.Y.); hehu.mech@csu.edu.cn (H.H.)

**Keywords:** microfluidic chip, ternary precursor, particle detection, hydrodynamic focusing

## Abstract

The microstructure of ternary precursors significantly influences the electrochemical performance of ternary cathode materials and, consequently, the overall performance of lithium-ion batteries. In industrial production utilizing traditional co-precipitation methods, Scanning Electron Microscopy (SEM) is typically employed for particle detection. However, this approach is limited by offline sampling lag, poor representativeness, cumbersome sample preparation, and low efficiency, failing to achieve real-time quality feedback on production lines. To enable high-throughput particle detection, this study proposes a multi-layer PDMS chip designed for three-dimensional (3D) hydrodynamic focusing. The sheath fluid compressed the sample flow in horizontal and vertical directions, respectively, to form a flat ribbon flow passing through the detection area. High-fidelity raw images are captured for automated particle microstructure analysis. Firstly, a chemical pretreatment protocol was optimized to ensure stable precursor solution transport. Secondly, a three-layer composite microchannel featuring a sequential horizontal and vertical sheath-flow compression mechanism was designed, with its geometry optimized via Computational Fluid Dynamics (CFD) simulations. Subsequently, experimental optimizations of flow rate ratios were performed using sodium fluorescein, followed by validation with ternary precursor solutions. The results indicate that the microchannel achieves flattened monolayer focusing of randomly distributed precursor particles, compressing the sample stream height to approximately 15.44 μm, thereby maintaining the particle stream within the microscope’s depth of field and analyzing particle microstructure efficiently based on a microscopic image. Moreover, it is confirmed that the focused stream dimensions are primarily governed by the flow rate ratio, allowing for a flexible increase in detection throughput by adjusting the total flow rate. Different from static offline particle analyzers, this platform captures dynamic particle morphology under continuous flow, providing real-time data to guide co-precipitation reaction adjustment.

## 1. Introduction

Ternary cathode materials are regarded as ideal candidates for lithium-ion batteries due to their high energy density. However, structural instability during cycling remains a critical bottleneck for their large-scale commercialization. The microstructure of ternary precursor particles, including primary particle morphology [1,2,3], particle size distribution and sphericity of secondary particles [2,4,5,6,7], and tap density [4,8,9,10], significantly influences the electrochemical performance of the resulting cathode materials. In industrial continuous precursor co-precipitation, particle size and morphology are monitored until approximately 12 μm for real-time reaction parameter adjustment, synthesizing highly radially aligned secondary particles for high-performance cathodes [2]. Consequently, the characterization of ternary precursor particles is indispensable for the optimization of precursor synthesis. In current industrial production, particle detection typically involves sampling from the reactor, followed by filtration, washing, and drying. Subsequently, Scanning Electron Microscopy (SEM) is utilized for microstructure analysis, while laser diffraction is employed to determine the particle size distribution. For instance, during the production of NCM precursors, Seo et al. [6] utilized SEM to collect microscopic images of precursor particles at 1, 3, 7, 15, and 18 h to observe morphological evolution, thereby adjusting reaction conditions to achieve high-quality precursors for mass production.

While SEM techniques provide high-resolution images of precursor particles and laser diffraction instruments offer particle size distribution data, traditional industrial detection methods are constrained by several limitations. These limitations include poor sampling representativeness, cumbersome sample preparation, the offline nature of the detection process, and a heavy reliance on time-consuming manual analysis. These factors significantly hinder the efficiency of rapid quality feedback in industrial-scale production. Consequently, to achieve real-time, online, and high-fidelity monitoring of precursor particles, there is an urgent need for a detection scheme capable of high throughput, dynamic observation, and automated image processing. Currently, imaging-based detection technologies for particles and cells have been extensively investigated. Microscopic imaging captures sequences of images with high temporal and spatial resolution, enabling the direct visualization of microscopic entities [11]. Haiden et al. [12] developed a system integrating microfluidic chips with dark-field microscopic imaging, utilizing Brownian motion tracking to achieve nanoscale size measurements of metal wear particles for early-warning applications. Zhu et al. [13] proposed SPLASH, an intelligent system combining polarization imaging, holography, and machine learning to achieve high-precision, non-invasive microplastic identification and distinction from natural particles. Huo et al. [14] developed an in situ measurement method based on a dual-view microscopic imaging system and 3D geometric modeling, utilizing image processing techniques to enhance image quality and reconstruct 3D particle geometries. Furthermore, Li et al. [15] introduced an innovative approach combining deep learning with Mueller matrix microscopic imaging for the identification of suspended particles. These studies demonstrate the application of microscopic imaging detection techniques in particle identification and morphological reconstruction. Nevertheless, such methods mainly apply to static or low-speed flows and well-monodispersed samples for easier characterization. For industrial ternary precursor slurry with high solid concentration, high particle density, and strong agglomeration tendency, particle overlapping and motion blur under dynamic flow seriously impede detection accuracy and throughput.

The hydrodynamic focusing (HDF) technique is a particle manipulation technique that can orderly arrange particles under continuous flow, which matches the demand of high-throughput dynamic imaging. Studies have explored the detection of biological cells and synthetic microparticles leveraging 3D hydrodynamic focusing. For instance, Du et al. [16] utilized bright-field imaging flow cytometry to capture images of flowing cells within wide channels, employing the YOLO-V4 deep neural network for cell type identification. Storti et al. [17] developed a fused silica 3D flow-focusing microfluidic device achieving compact, high-throughput, and size-independent focusing. Their system enabled label-free monitoring of *Escherichia coli* bacteria by capturing bacterial transit signals. Eluru et al. [18] proposed a 3D flow-focusing device fabricated via single-layer polydimethylsiloxane (PDMS) replication molding. Featuring two sheath inlets, the device demonstrated stable 3D focusing even under highly variable sample flow rates. Furthermore, Zhou et al. [19] designed and validated a novel microfluidic cytometer based on a 3D hydrodynamic focusing mechanism, which utilized adaptive sheath flow to achieve precise sample focusing, significantly enhancing the signal-to-noise ratio and measurement accuracy of particle impedance signals. Existing microfluidic studies on particle detection predominantly utilize standard polystyrene (PS) microspheres [20,21,22,23], biological cells [18,22,24,25], and bacteria [17], whose density (1.03 to 1.12 g/cm^3^) is close to the carrier fluid. While these studies provide valuable methodological and technical frameworks for the application of microfluidics in particle detection, implementing such schemes specifically for ternary precursor particles presents unique and significant challenges.

Challenge 1: Particle agglomeration and sedimentation induced by high density and van der Waals interparticle forces, which easily disturb the flow field distribution. Ternary precursor particles have a higher density of approximately 2 g/cm^3^, which differs from the above samples.

Challenge 2: Geometric regulation of microchannel on a flattened monolayer focused stream. Previous studies have primarily focused on constraining particles into single-file sequences for detection [18,21,24,26]. Alternative schemes adopted ultra-shallow microchannels to confine particle streams vertically via wall limitation [12,27]. However, High-throughput precursor particle detection relies on a flattened monolayer focused stream, eliminating vertical particle overlap, which imposes stringent geometric design requirements on the channel structure.

Challenge 3: Synergistic regulation of focused stream dimensions and spatial positioning via flow rate parameters. Once the microchannel is established, flow rate parameters determine optimal focused stream and high-throughput detection. The focused stream with maximum lateral width and vertical thickness of approximately 1.5 particle diameters flows in a wide channel, which brings flow velocity gradients [28,29] and vertical offsets of the focused stream. To ensure that parallel particle streams remain within the optimal focal height range, flow rate parameters must be adjusted with high precision.

To address these challenges, this study develops an integrated platform combining hydrodynamic focusing and microscopic image analysis for the high-throughput and stable detection of precursor particles. Microfluidic chip design and sample dispersion strategies are optimized, and the effects of critical parameters on particle focusing performance are systematically investigated, providing a solid data basis for subsequent image processing. We propose a three-dimensional (3D) hydrodynamic focusing device constructed from multiple layers of bonded polydimethylsiloxane (PDMS). Featuring two sheath inlets, the device achieves 3D hydrodynamic focusing through sequential horizontal and vertical compression. This mechanism ensures that precursor particles are arranged in a monolayer as they pass through the detection zone, where a high-speed camera captures high-contrast microscopic images for downstream analysis. Finally, the focusing performance was experimentally validated, and the device was successfully applied to the detection of pretreated ternary precursor samples.

## 2. Materials and Methods

### 2.1. Preparation of the Sample

The detection subject of this study is a ternary precursor solution collected from the reactor in the laboratory. Raw ternary precursor slurry from the reactor suffers severe particle agglomeration and sedimentation, alongside a risk of clogging microchannels. To ensure compatibility between the ternary precursor solution and the operational requirements of the microfluidic chip, sample pretreatment using dispersant and thickener was performed.

After screening tests detailed in Appendix A, 0.6% CMC was selected as the optimal thickener. The pretreated sample achieved a Zeta potential of −30 mV and viscosity of 15 mPa·s, providing a long-term stable suspension for particle detection. In 3D hydrodynamic focusing, mismatched physicochemical properties between sample and sheath fluid affect focusing performance. The sheath fluid must be chemically inert relative to the sample, and precise refractive index matching between the two fluids is essential for high-fidelity optical detection. Consequently, a 40% glycerol (Sinopharm Chemical Reagent Co., Ltd., Shanghai, China) -water solution was formulated as the sheath fluid to closely match the density and viscosity of the sample solution, thereby minimizing diffusion and suppressing gravitational sedimentation.

Furthermore, prior to utilizing the ternary precursor solution as the sample, preliminary validation of the 3D hydrodynamic focusing experiments was conducted using alternative materials with relatively simpler physicochemical properties. Specifically, a 0.5 mM sodium fluorescein (Macklin Biochemical Co., Ltd., Shanghai, China) solution was prepared as the sample, while deionized water was utilized as the sheath fluid. During these experiments, fluorescence images of the focused flow were captured to evaluate the performance and stability of the 3D hydrodynamic focusing.

### 2.2. Design of the Channel

Pretreatment of the ternary precursor solution yields a well-dispersed suspension which enables effective hydrodynamic focusing. Stable monolayer particle streams within the micro-imaging region are required to accurately analyze particle microstructures. Various micro-channel designs can realize particle focusing and detection. However, these structures fail to meet the requirements for 3D hydrodynamic focusing of ternary precursor particles in this work. The precursor particles have high density and relatively broad size distribution, which impose stringent demands on the intensity and uniformity of the sheath flow. In addition, clear microscopic imaging is only achievable for particles located within the camera’s depth of field. Therefore, the focused stream must be precisely controlled along the direction perpendicular to the focal plane. A dedicated micro-channel is thus developed to achieve high-throughput focusing of ternary precursor particles.

**Figure 1:**
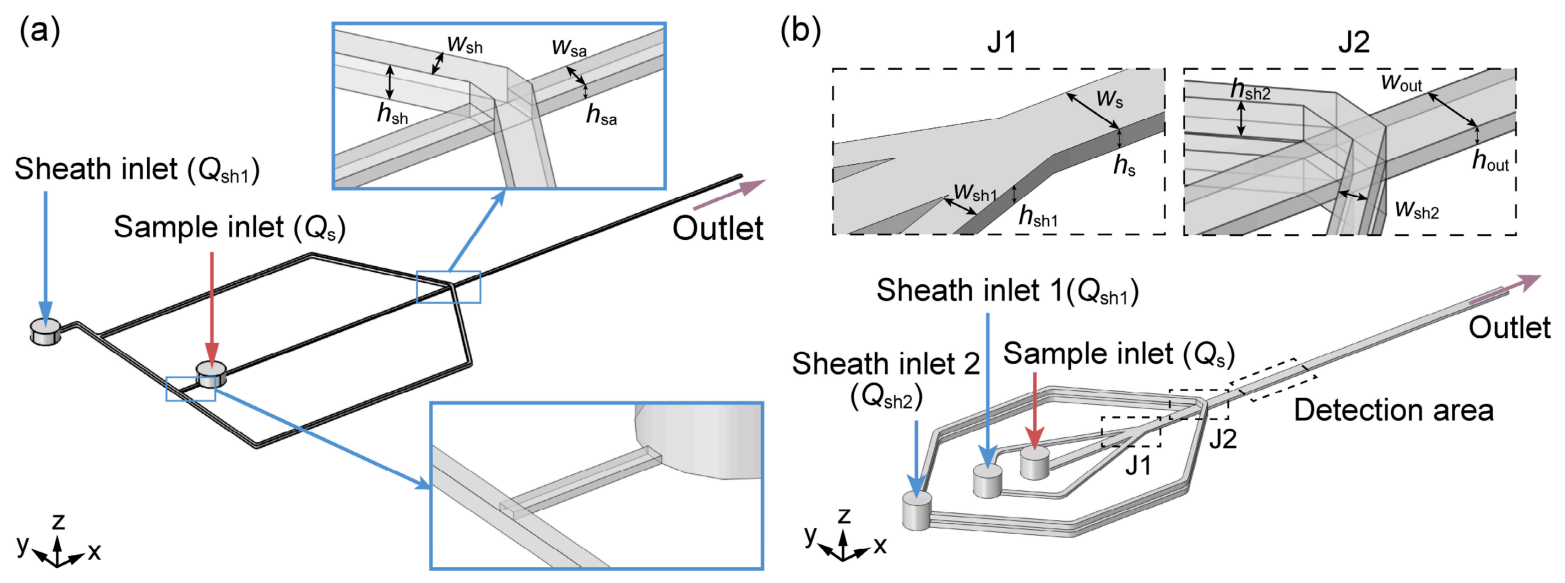
Schematic of the microfluidic chips. (**a**) Channel A. (**b**) Channel B.

Two micro-channels were designed, as shown in the schematic in Figure 1. The design incorporates moderate horizontal compression to prevent particle adhesion to the channel walls, while applying enhanced vertical compression to highly constrain the sample flow to a height approaching the diameter of a single particle. Microchannel A features one sample inlet and one sheath fluid inlet, with main channel dimensions of $w_{\mathrm{sa}}=100\;\mu m$ in width and $h_{\mathrm{sa}}=40\;\mu m$ in height, and sheath channel dimensions of $w_{\mathrm{sh}}=100\;\mu m$ in width and $h_{\mathrm{sh}}$ in height. The sheath fluid is injected into the main channel and split into two streams, compressing the sample to form a focused stream near the channel bottom.

Microchannel B consists of one sample inlet and two sheath fluid inlets. The main channel has a width of $w_{s}=200\;\mu m$ and a height of $h_{s}=50\;\mu m$. For the sheath channels, the horizontal focusing channel has a width of $w_{\mathrm{sh}1}$ and a height of $h_{\mathrm{sh}1}=50\;\mu m$, while the vertical focusing channel has a width of $w_{\mathrm{sh}2}=100\;\mu m$ and a height of $h_{\mathrm{sh}2}$. The horizontal sheath fluid first merges with the main channel at junction J1 to compress the sample flow and achieve horizontal focusing. Subsequently, the vertical sheath fluid is injected at junction J2 to achieve vertical focusing, thereby forming a 3D focused stream. Theoretically, both microchannel designs are capable of confining the sample flow to the center of the channel. While Microchannel A is easier to fabricate and has fewer sheath inlets, Microchannel B, despite its higher structural complexity, allows for the independent regulation of sheath flow rates in horizontal and vertical directions.

### 2.3. Numerical Simulation

The dimensions of the focused stream are governed by the dimensions of the channel. The key structural parameters include: the ratio of the sheath-to-sample channel height for Channel A $R_{\mathrm{ha}}=h_{\mathrm{sh}}/h_{\mathrm{sa}}$; the width ratio of the horizontal focusing sheath channel to the sample channel for Channel B $R_{w}=w_{\mathrm{sh}1}/w_{s}$; the height ratio of the vertical focusing sheath channel to the sample channel $R_{h}=h_{\mathrm{sh}2}/h_{s}$; and the intersection angle α between the horizontal focusing sheath channel and the sample channel. Furthermore, flow rate parameters significantly impact the focusing performance. These include the sheath-to-sample flow rate ratio for Channel A $R_{\mathrm{va}}=Q_{\mathrm{sh}}/Q_{\mathrm{sa}}$, and for Channel B, the horizontal focusing sheath-to-sample flow rate ratio $R_{v1}=Q_{\mathrm{sh}1}/Q_{s}$ and the vertical focusing sheath-to-sample flow rate ratio $R_{v2}=Q_{\mathrm{sh}2}/Q_{s}$. Consequently, to determine the optimal channel dimensions and flow rate parameters for achieving hydrodynamic focusing of precursor particles, computational fluid dynamics (CFD) simulations will be performed to analyze the influence of these critical parameters on the focusing performance.

Computational Fluid Dynamics (CFD) was employed using COMSOL Multiphysics 6.2 to simulate and analyze the internal flow field of the microchannel and the particle trajectories under hydrodynamic focusing. The software’s built-in Laminar Flow module was utilized to simulate the process where the sheath fluid compresses the sample solution to form a focused stream. Given that the Reynolds number (Re) within the microchannel is significantly lower than 2300, the flow remains in a laminar state, governed by the Navier–Stokes equations. The viscosity and density for both the sample and sheath fluids were set to $\mu_{s}=15\;\mathrm{mPa}\cdot s$ and $\rho_{s}=1300\;\mathrm{kg}/m^{3}$, respectively. The Particle Tracing for Fluid Flow module was selected to solve for particle trajectories within the flow field, with particles defined by a diameter of 10 μm and a density of $2000\;\mathrm{kg}/m^{3}$. Forces such as drag and gravity were taken into account. Regarding boundary conditions, particle-wall interactions were set to Bounce, and particles were set to Disappear upon reaching the outlet. Fluid inlets were configured as velocity inlets with specified flow rates, while the outlet was defined as a pressure outlet with a relative pressure of 0 Pa. A no-slip condition was applied to the channel walls.

The mesh was configured as a user-controlled mesh, incorporating corner refinement and boundary layer settings. A mesh independence verification was performed on the established model, with the results for Channel B presented in Table 1. As the parameter a decreases, the average relative error of the maximum cross-sectional velocity remains within 3%. This indicates that the meshing scheme at a = 1 provides numerical solutions with sufficient accuracy. At this configuration, the total number of mesh elements is 288,639, and further refinement would lead to a substantial increase in the total element count. Balancing computational accuracy with hardware load, the mesh configuration at a = 1 was selected for subsequent simulations.

Parametric simulations were conducted to investigate the effects of structural parameters ($R_{w}$, $R_{h}$, α) and flow rate parameters ($R_{v1}$, $R_{v2}$) on the focused stream dimensions, with simulation settings detailed in Table 2 and Table 3, respectively.

### 2.4. Fabrication of Device

**Figure 2:**
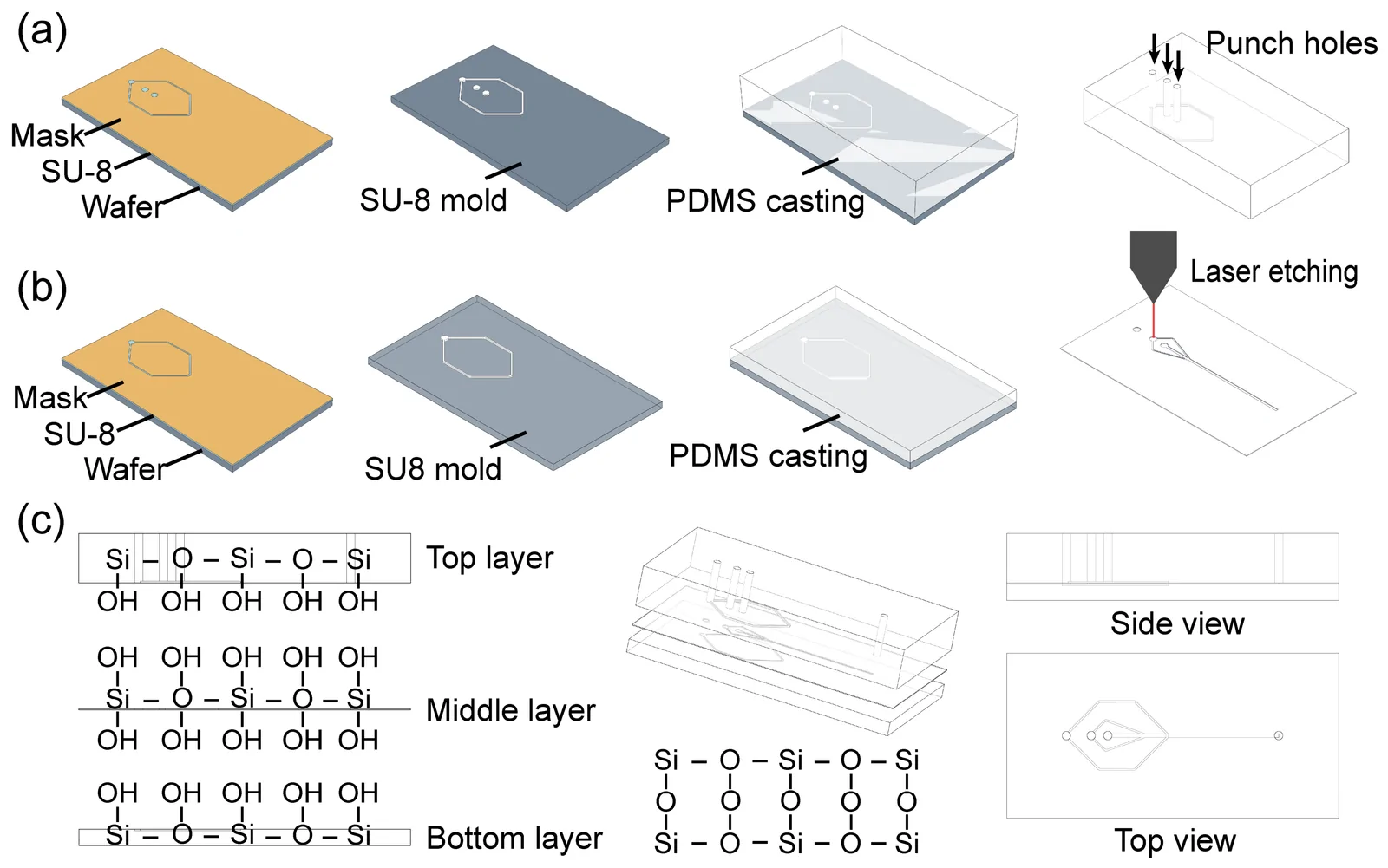
Fabrication procedure of the PDMS chip. (**a**) Fabrication procedure of the top PDMS layer; (**b**) Fabrication procedure of the middle PDMS layer; (**c**) Bonding of PDMS layers.

In the fabrication of traditional PDMS chips, the primary technical challenge lies in the alignment precision required during the bonding of multiple PDMS layers. In this design, Channel B consists of three layers: the middle layer containing the main channel and horizontal focusing sheath channels, and the upper and lower layers containing the vertical focusing sheath channels. The critical alignment occurs at the junction where the vertical sheath channels intersect the main channel. However, because the channel segment at the intersection is relatively short, the requirements for alignment precision are effectively reduced. Furthermore, due to the small height of the middle layer, which is significantly challenging for soft lithography, laser etching was employed for its fabrication, while the upper and lower layers were processed using soft lithography. The final assembly involved aligning the three layers under a microscope, followed by bonding. To achieve high-quality microscopic observation, the bottom PDMS layer was set to a thickness of 1 mm, and the top layer was made 3 mm thick to ensure the steel pins inserted into the chip inlets stably. The complete fabrication procedure is illustrated in Figure 2.

### 2.5. Experimental Setup and Procedure

**Figure 3:**
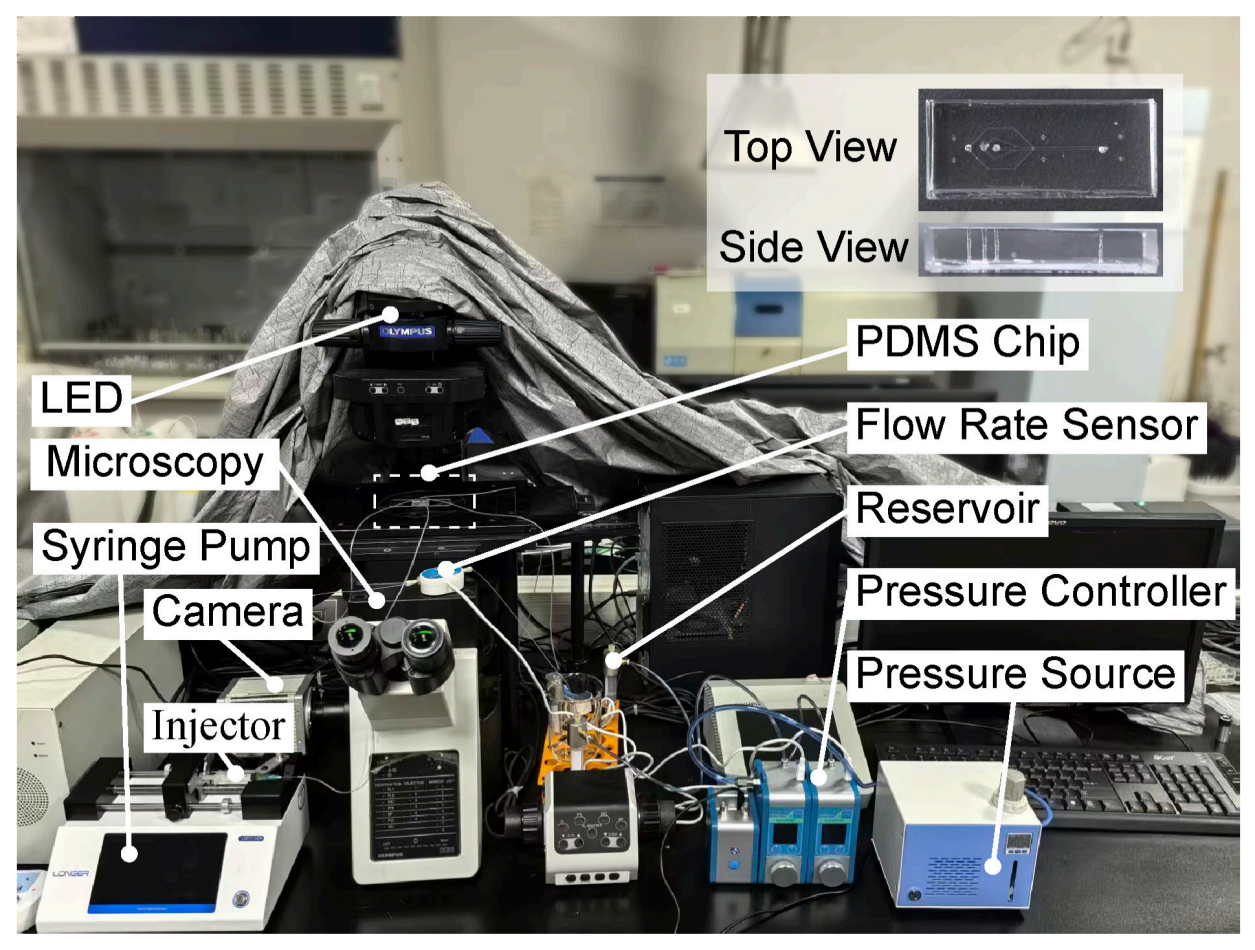
Microfluidic hydrodynamic focusing experimental platform.

To validate the proposed design and analyze the performance of hydrodynamic focusing where the sample stream is sheathed by the surrounding fluid, experiments were conducted. As illustrated in Figure 3, a microfluidic experimental platform was established. The platform includes an inverted microscope (IX83, Olympus, Tokyo, Japan) equipped with an Olympus 20X objective (0.45 NA, Evident Scientific, Tokyo, Japan) and two cameras: a Hamamatsu ORCA-Flash4.0 (C11440-42U, Hamamatsu Photonics K.K., Hamamatsu, Japan) and a high-speed camera (SH3-108, SinceVision, Shenzhen, China). A PDMS chip, a pressure pump with an adjustable range: 0~2000 mbar (Fluigent Flow EZ, Fluigent, Paris, France), a syringe pump (LSP11-1AP, Longer Precision Pump Co., Ltd., Baoding, China), and a microfluidic flow sensor (FLOW UNIT, Fluigent, Paris, France). Additionally, reservoirs for both sample and sheath fluids and disposable syringes were used. Using this experimental platform, top-view and side-view images of the focused flow within the microchannels were captured to evaluate the 3D hydrodynamic focusing performance of the PDMS chip. Before each experiment, the microchannels were primed with sheath fluid to expel any residual air. It is critical to ensure no air bubbles are trapped on the channel walls, as they can alter the local flow field and affect the stability of the focusing process.

The first set of experiments investigated how the horizontal sheath-to-sample flow rate ratio affects the focused stream dimensions. A 0.5 mM sodium fluorescein solution was used as the sample and injected into the sample inlet at a flow rate of 6 μL/min. Deionized water served as the sheath fluid, with the flow rate at 36 μL/min. The horizontal focusing flow rate ratio, $R_{v1}$ (defined as the ratio of the horizontal sheath flow rate to the sample flow rate, $Q_{\mathrm{sh}1}/Q_{s}$), was varied across 0.25, 0.5, 0.75, 1, 1.25. Fluorescence images of the stream were collected from a top-view perspective for subsequent analysis.

The second set of experiments investigated how the vertical sheath-to-sample flow rate ratio affects the focused stream dimensions. A 0.5 mM sodium fluorescein solution was used as the sample and injected into the sample inlet at a flow rate of 6 μL/min. Deionized water served as the sheath fluid, with the horizontal focusing sheath flow rate maintained at 3 μL/min. The vertical focusing flow rate ratio, $R_{v2}$ (defined as the ratio of the vertical sheath flow rate to the sample flow rate, $Q_{\mathrm{sh}2}/Q_{s}$), was set to 2, 4, 6, 8, and 10, respectively. Fluorescence images of the stream were collected from both top-view and side-view perspectives for analysis.

The third set of experiments investigated how the total flow rate affects the focused stream dimensions. A 0.5 mM sodium fluorescein solution served as the sample, with the sample flow rates ($Q_{s}$) set at 2, 4, 6, 8, and 10 μL/min. Deionized water was used as the sheath fluid, while the flow rate ratios were maintained at $R_{v1}=0.5$, $R_{v2}=6$. Fluorescence images of the stream were collected from both top-view and side-view perspectives for analysis.

The fourth set of experiments evaluated the focusing performance of the microchannel for precursor particles. The pretreated ternary precursor solution was injected as the sample at a flow rate of 6 μL/min. With deionized water as the sheath fluid, the sheath flow rate ratio $R_{v2}$ was varied across 6, 8, and 10. Top-view and side-view images of the precursor particles were collected for subsequent analysis.

**Figure 4:**
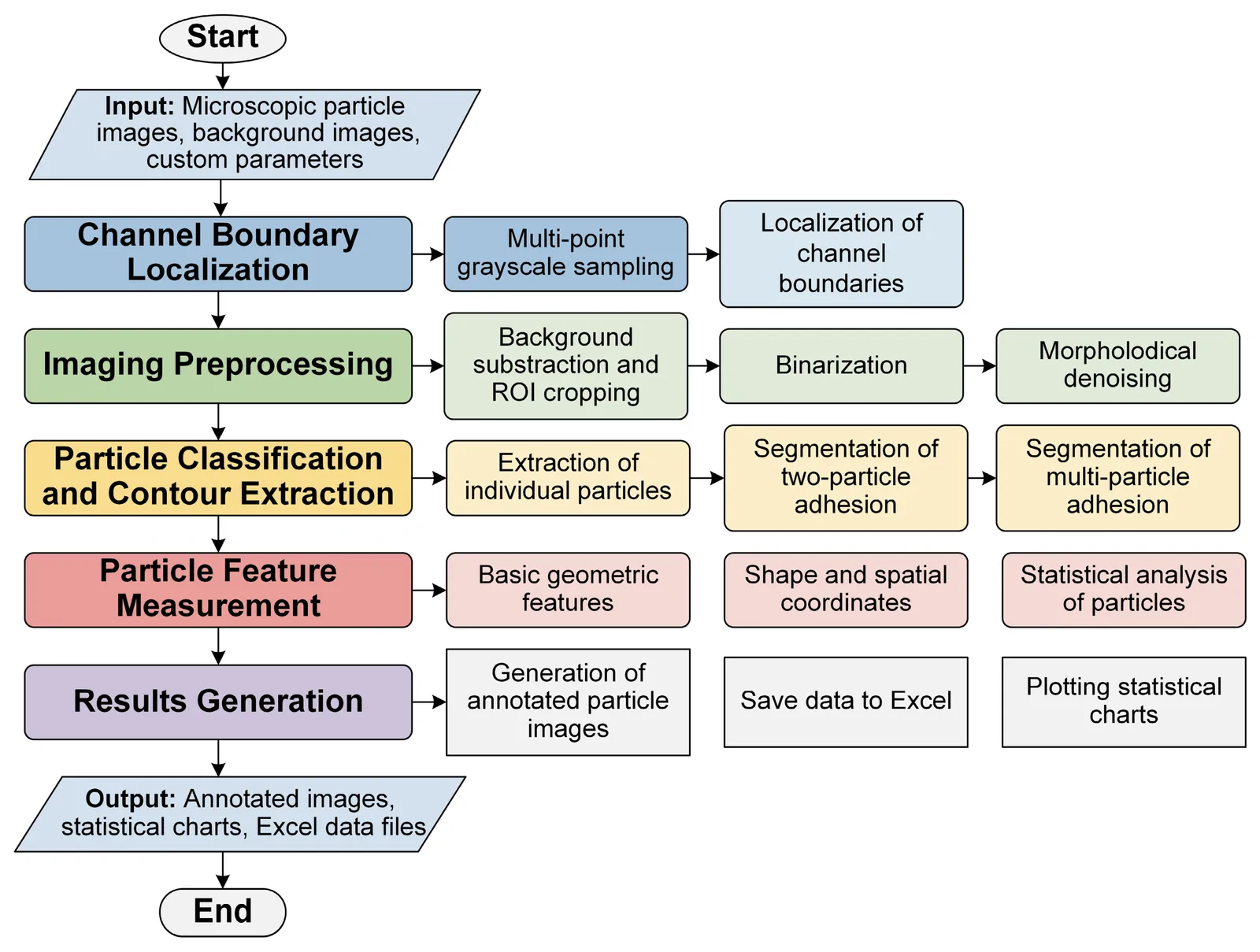
Precursor particle image analysis workflow.

For the top-view precursor particle images obtained from the fourth experimental group, an integrated image processing algorithm was employed. This algorithm facilitates microchannel boundary localization, image preprocessing, particle segmentation, and feature measurement, thereby enabling automated image-based analysis of particle characteristics. The detailed workflow is illustrated in Figure 4.

## 3. Results and Discussion

### 3.1. Effect of Structural Parameter on Hydrodynamic Focusing

**Figure 5:**
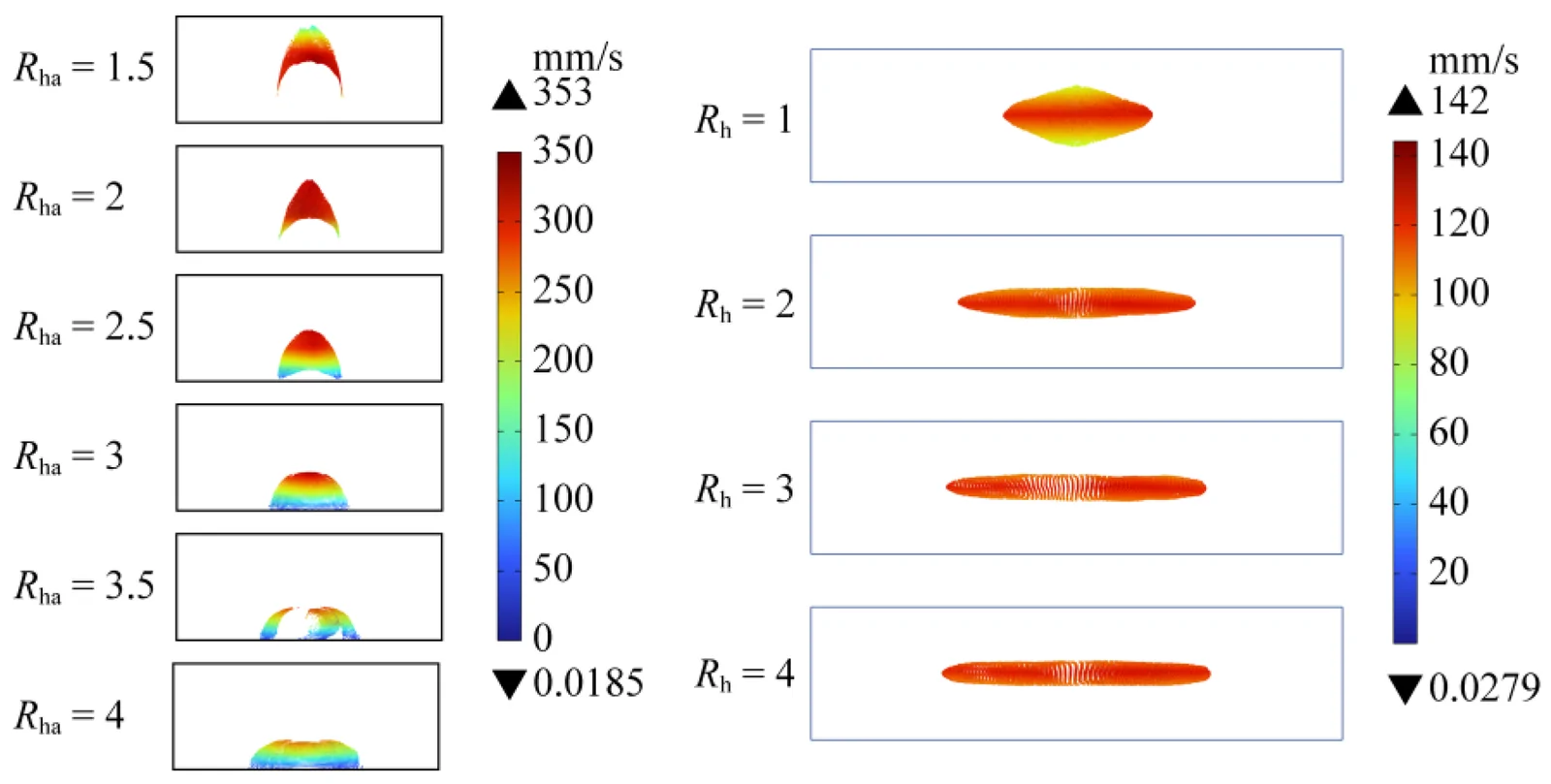
Simulated outlet cross-sectional streamlines for the two designs at various channel height ratios.

Based on the simulation settings detailed in Section 2.3, results were obtained for various structural parameters to evaluate the hydrodynamic focusing performance. Figure 5 shows the streamlines at the outlet cross-sections of the two microchannels under different height ratios. The focused stream in Channel B more closely approximates a flattened monolayer shape, which is consistent with the intended focusing flow shape.

**Figure 6:**
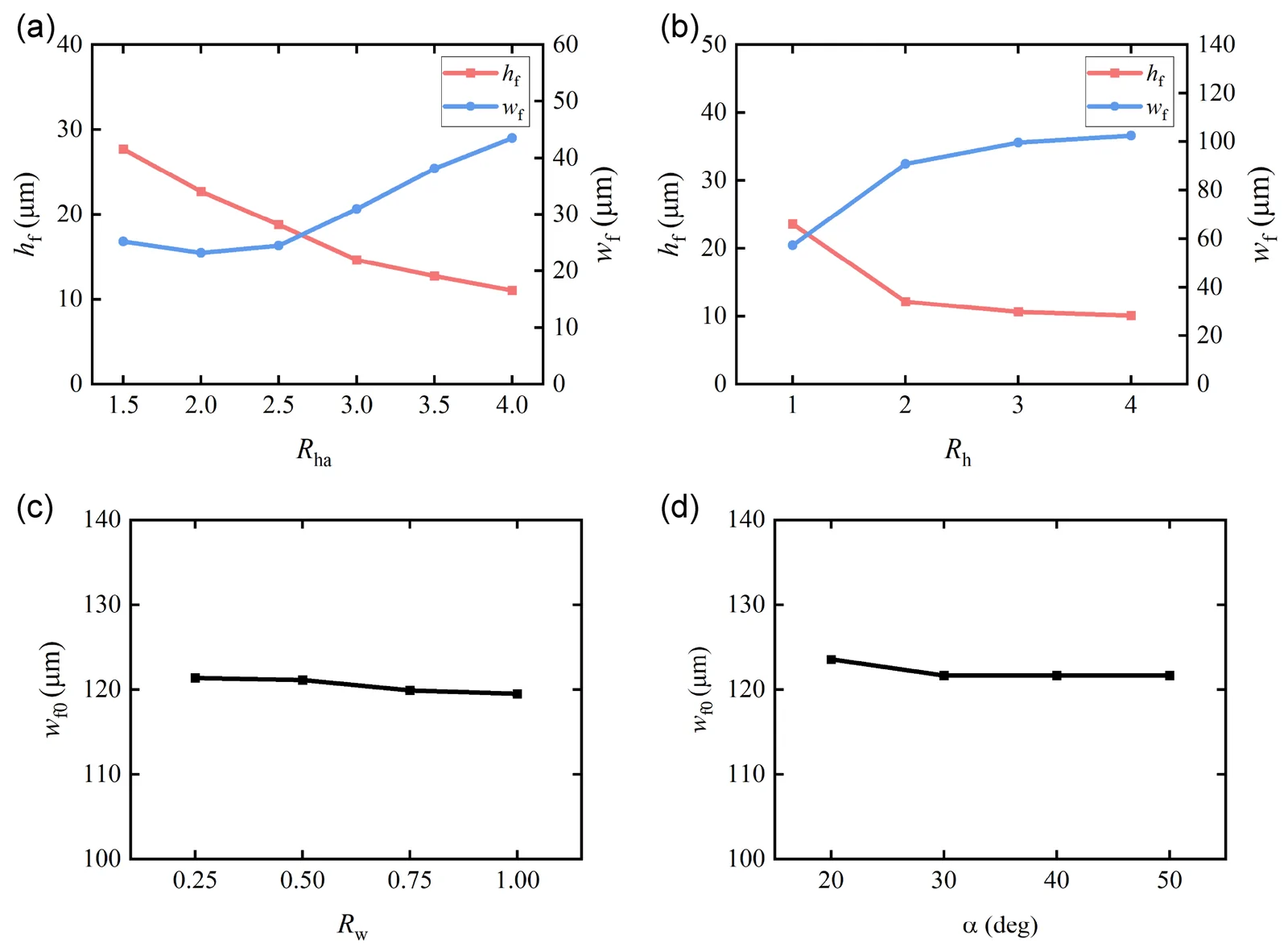
Simulated focused stream dimensions under different structural parameters. (**a**) Channel A at different height ratios $R_{\mathrm{ha}}$. (**b**) Channel B at different height ratios $R_{h}$. (**c**) Channel B at different width ratios $R_{w}$. (**d**) Channel B at different channel angles α.

The dimensions of the focused stream, measured from the simulated streamlines under various structural parameters, are presented in Figure 6. All simulation data were visualized and plotted using OringinPro 2021. For Channel A, when the height ratio $R_{\mathrm{ha}}=3$, the focused stream height is approximately 13.31 μm, which is nearly 1.5 times the diameter of the precursor particles. While this configuration achieves effective vertical focusing of the sample stream, the focused stream is positioned near the channel bottom. Regarding Channel B, as $R_{h}>2$, the reduction in focused stream height and the increase in focused stream width both become slight. Therefore, a channel height ratio of $R_{h}=2$ was selected.

As the channel width ratio $R_{w}$ increases, there is little variation in the primary focused stream width $w_{f0}$. To prevent the sample stream from contacting the channel walls while maintaining high sample throughput and ensuring ease of fabrication, a width ratio of $R_{w}=0.5$ was chosen. Finally, since the primary focused stream width $w_{f0}$ is relatively insensitive to changes in the channel intersection angle, an angle of α = 20° was selected. This choice accounts for the adhesion and sedimentation tendencies of the particles, providing a larger channel dimension at the junction to reduce the risk of clogging.

### 3.2. Effect of $R_{v1}$ ($Q_{\mathrm{sh}1}/Q_{s}$) on Horizontal Hydrodynamic Focusing

**Figure 7:**
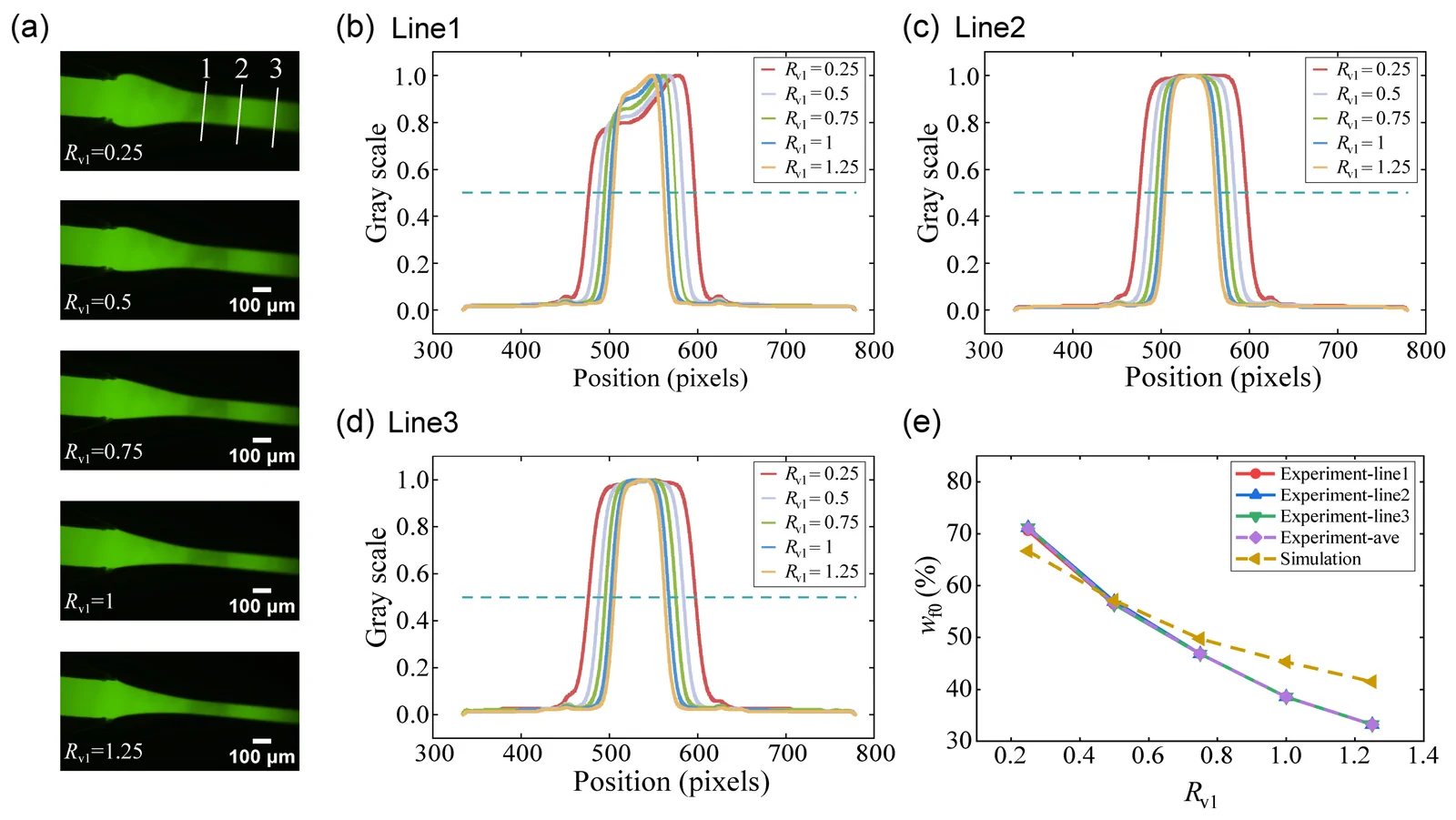
Analysis of horizontal focusing performance of the fluorescent focused stream. (**a**) Top-view fluorescence images of the focused stream at junction J1 under different horizontal focusing sheath-to-sample flow rate ratios $R_{v1}$. (**b**) Normalized grayscale intensity profiles of the focused stream at Line 1. (**c**) Normalized grayscale intensity profiles of the focused stream at Line 2. (**d**) Normalized grayscale intensity profiles of the focused stream at Line 3. (**e**) Initial focused stream width $w_{f0}$ obtained from experiments and simulations.

Figure 7a shows fluorescence microscopic images of the focused stream at J1. As the horizontal flow rate ratio $R_{v1}$ increased, the fluorescent stream is significantly compressed in the horizontal direction. For quantitative analysis, normalized gray scale intensity distributions were extracted along vertical analysis lines (Line 1, Line 2, and Line 3), as illustrated in Figure 7b–d. The width of the primary focused stream, $w_{f0}$, is defined as the full width at half maximum (FWHM) of the gray scale intensity curve. Figure 7e presents the variation in the primary focused stream width ($w_{f0}$) as a function of the flow rate ratio for both simulation and experimental results.

The experimental trends are consistent with the simulation, showing that $w_{f0}$ decreases as $R_{v1}$ increases. However, at lower flow rate ratios ($R_{v1}=0.25$ and 0.5), the experimental width is higher than the simulated value. This discrepancy is primarily attributed to the following factors: firstly, the laser etching process introduces deviations between the actual dimensions of the PDMS chip and the simulation model, alongside microchannel wall roughness; secondly, the molecular diffusion of the fluorescent tracer leads to blurred boundaries of the focused stream. Overall, the results indicate that the optimal horizontal flow rate ratio is $R_{v1}=0.5$. This configuration prevents the sample stream from contacting the channel sidewalls while maintaining a maximum focused width to satisfy high-throughput requirements. At this ratio, the focused stream is compressed to approximately 57% of the total channel width.

It should be noted that sodium fluorescein solution has density and viscosity much closer to pure water, which differs from the ternary precursor slurry. The sodium fluorescein solution presents laminar diffusion without particle sedimentation, while precursor particles produce slight vertical offset under gravity. Therefore, the vertical sheath flow rate ratio is further optimized to compensate for vertical offset in particle experiments.

### 3.3. Effect of $R_{v2}$ ($Q_{\mathrm{sh}2}/Q_{s}$) on Hydrodynamic Focusing

**Figure 8:**
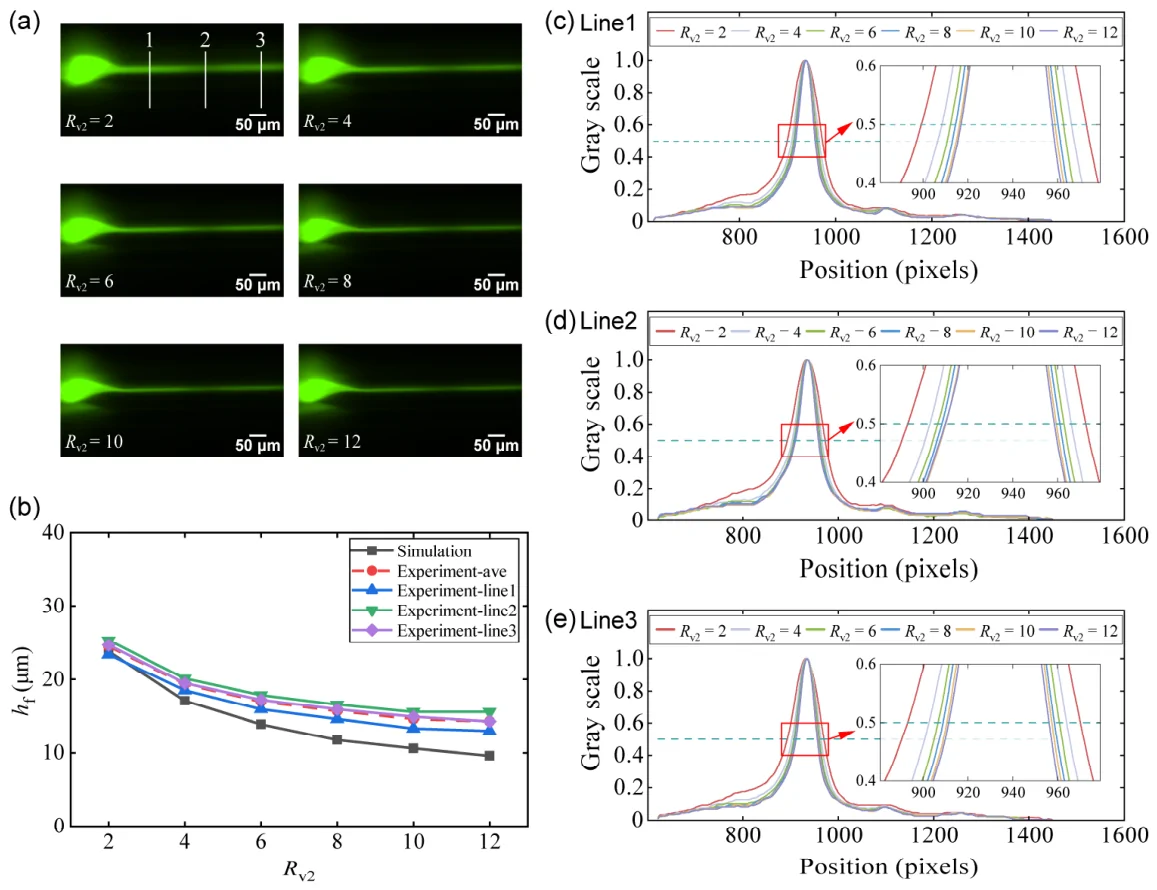
Analysis of vertical focusing performance of the fluorescent focused stream. (**a**) Side-view fluorescence images of the focused stream downstream of junction J2 under different vertical focusing sheath-to-sample flow rate ratios $R_{v2}$. (**b**) Focused stream height $h_{f}$. obtained from experiments and simulations. (**c**) Normalized grayscale intensity profiles of the focused stream at Line 1. (**d**) Normalized grayscale intensity profiles of the focused stream at Line 2. (**e**) Normalized grayscale intensity profiles of the focused stream at Line 3.

Figure 8a shows fluorescence microscopic images of the focused stream at J2. As the vertical flow rate ratio ($R_{v2}$) increased, the fluorescent stream was compressed more significantly. To quantify this effect, vertical analysis lines (Line 1, Line 2, and Line 3) were defined in the images to extract and normalize the fluorescence gray scale distributions, as shown in Figure 8c–e. The experimental height of the focused stream ($h_{f}$) is defined as the distance corresponding to the full width at half maximum (FWHM, where the normalized gray scale value is 0.5) of the intensity curve.

The experimental results demonstrate a consistent trend with the simulations, with $h_{f}$ decreasing as $R_{v2}$ increases. Across the three analysis lines, the measured stream heights closely approximate the simulated values. However, at $R_{v2}>4$, the experimental heights are slightly higher than those predicted by the simulation. Furthermore, as the focused stream develops along the flow direction, variations in $h_{f}$ are observed at the positions of Line 1, Line 2, and Line 3 across different flow rate ratios, with a standard deviation within 1.3 μm. This is attributed to minor pressure fluctuations within the PDMS chip, causing slight variations in the stream height along the flow path while maintaining an overall narrow stream profile. The comprehensive results indicate that to achieve a focusing height of approximately 1.5 times the particle diameter, the optimal flow rate ratio is $R_{v2}=8$ (corresponding to $Q_{s}=6\;\mu L/\min$, $Q_{\mathrm{sh}1}=3\;\mu L/\min$, $Q_{\mathrm{sh}2}=48\;\mu L/\min$), at which the focused stream is compressed to a height of 15.69 μm.

**Figure 9:**
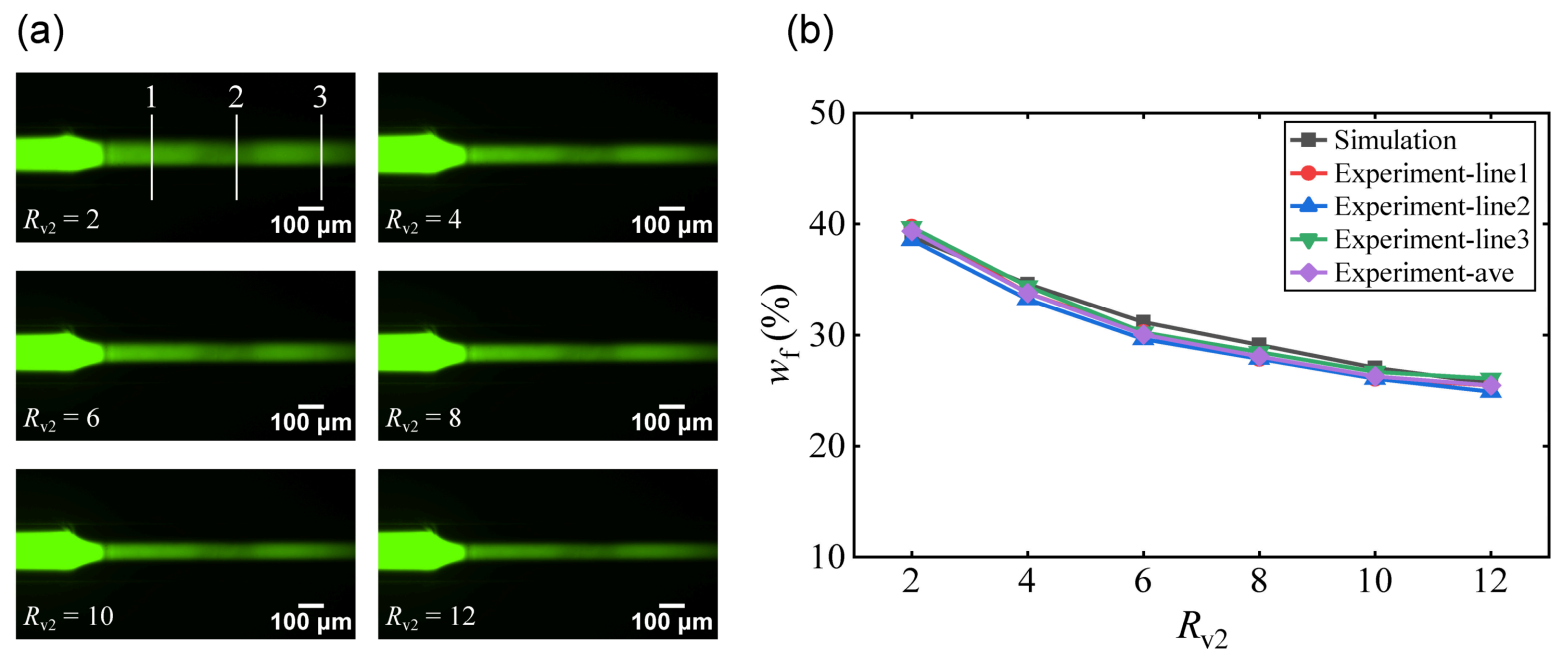
Analysis of horizontal focusing performance of the focused stream in the detection region. (**a**) Top-view fluorescence images of the focused stream downstream of junction J2 under different flow rate ratios $R_{v2}$; numbers 1, 2 and 3 mark the three analysis lines selected for grayscale extraction. (**b**) Focused stream width in experiments and simulations.

To evaluate the potential for high-throughput detection, particularly regarding whether the sample stream width is sufficiently large, an analysis of the focused stream width was performed. Figure 9a displays a top-view fluorescence microscopic image of the focused stream recorded at J2. Analysis lines (Line 1, Line 2, and Line 3) were established at 40%, 65%, and 90% of the image width, respectively. The corresponding gray scale intensities were extracted and normalized, with the focused stream width defined at the point where the normalized gray scale value is 0.5. The percentage of the focused stream width relative to the total channel width under various flow rate ratios ($R_{v2}$) is presented in Figure 9b.

Across different $R_{v2}$ values, the experimentally measured width ratios show good agreement with the simulation results. At flow rates of $Q_{s}=6\;\mu L/\min$, $Q_{\mathrm{sh}1}=3\;\mu L/\min$, $Q_{\mathrm{sh}2}=48\;\mu L/\min$, the focused stream width occupies approximately 30.05% of the microchannel width (roughly 60 μm). Compared to traditional focusing methods where the stream width is limited to a single particle diameter, the focused stream generated by the chip design in this study is significantly wider, thereby enabling higher levels of particle throughput.

### 3.4. Effect of Total Flow Rate on Hydrodynamic Focusing

**Figure 10:**
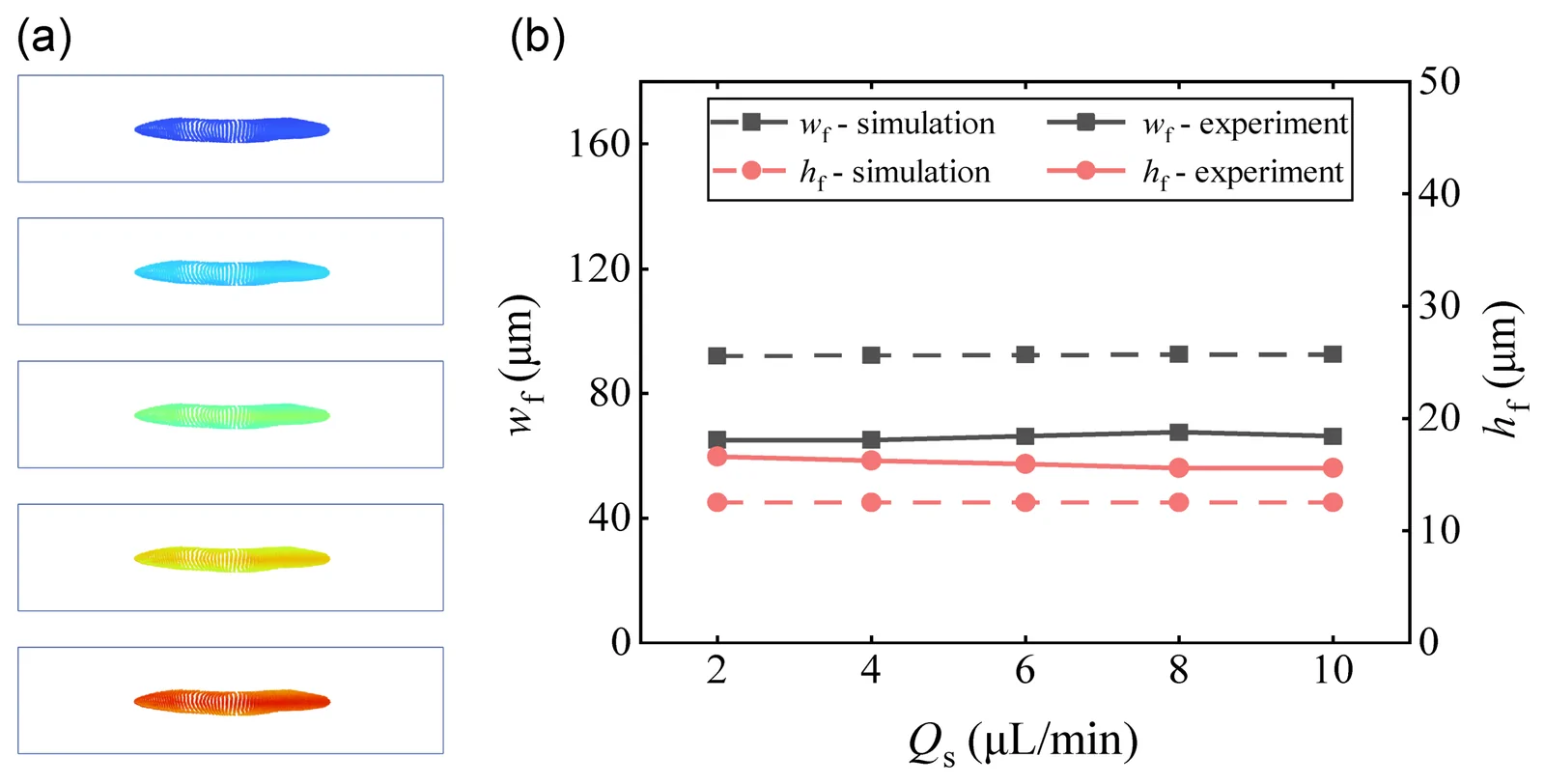
Analysis of focusing performance under different total flow rates at a fixed flow rate ratio based on simulations and experiments. (**a**) Cross-sectional streamlines at the channel outlet under different sample flow rates; different colors correspond to different flow rates. (**b**) Percentage of focused stream width relative to channel width and percentage of focused stream height relative to channel height.

Following the experimental setup described in Section 2.5, experiments were conducted to evaluate the dimensions of the focused stream across five distinct sample flow rates. Figure 10a displays the simulated streamline distributions at the channel cross-section under various flow rates, showing that the width and height of the focused stream remain nearly invariant with changes in the sample flow rate ($Q_{s}$). Figure 10b presents the widths and heights obtained from both experimental measurements and simulations. Overall, the observed curves are relatively plateaued, and the calculated mean values and standard deviations for the focused stream dimensions are summarized in Table 4. The minor standard deviations indicate that when the flow rate ratio is held constant, the magnitude of the total flow rate has a negligible influence on the dimensions of the focused stream.

The insensitivity of the focused stream dimensions to the total flow rate is primarily attributed to the laminar flow regime within the PDMS chip. Under the maximum sample flow rate of $Q_{s}=10\;\mu L/\min$, the Reynolds number (Re) remains below 1. Consequently, fluid motion is dominated by viscous forces, while the influence of inertial forces is negligible. In this regime, the spatial distribution of the sample stream is dictated by the relative velocity ratio between the sheath and sample fluids rather than the total flow rate. This interpretation is supported by the agreement between the experimental and simulated focused stream dimensions. These findings imply that in practical applications, the focused stream dimensions can be precisely controlled by adjusting the flow rate ratio, whereas the total flow rate can be adjusted to change particle velocity, thereby adapting diverse detection throughput requirements.

### 3.5. Precursor Particle Detection Experiments

**Figure 11:**
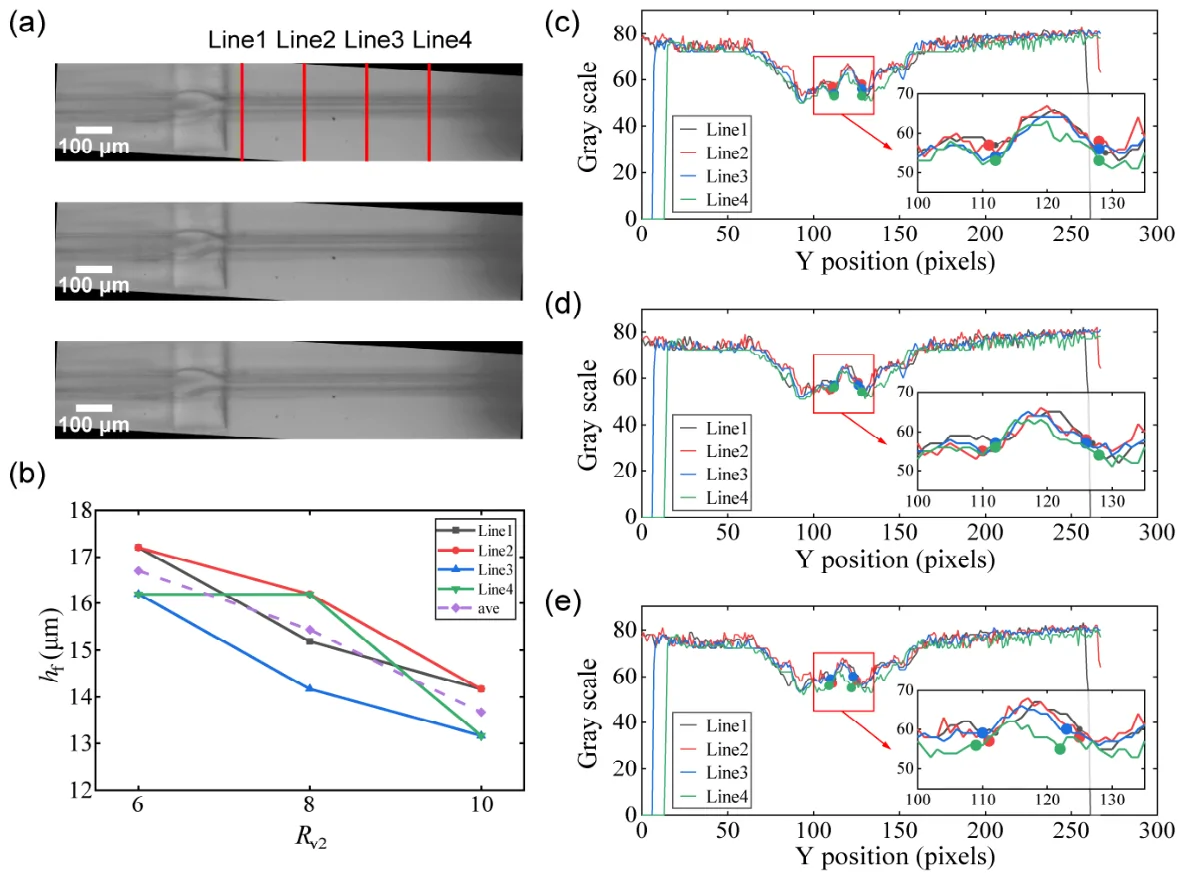
Analysis of vertical particle focusing performance under different vertical focusing sheath flow rates. (**a**) Side-view precursor images of the focused stream at J2 under different flow rate ratios $R_{v2}$. (**b**) Focused stream heights under different flow rate ratios $R_{v2}$. (**c**) Gray scale intensity profiles at flow rate ratio $R_{v2}=6$; coloured circles denote the two edge positions of the gray-scale peak used to calculate the vertical focusing height. (**d**) Gray scale intensity profiles at flow rate ratio $R_{v2}=8$. (**e**) Gray scale intensity profiles at flow rate ratio $R_{v2}=10$.

Following the experimental setup of the fourth group described in Section 2.5, the 3D hydrodynamic focusing effect of precursor particles was validated in the PDMS chip. Side-view images of the focused stream were collected in the xz-plane. Figure 11a shows the focused stream formed by the vertical compression of the sample flow containing precursor particles. Four vertical analysis lines were selected in the side-view images for grayscale intensity analysis, as shown in Figure 11c–e. Owing to the distinct light absorption properties of the sample and sheath fluid, the grayscale curves form characteristic central peaks, whose widths correspond to the vertical height of the focused stream.

The results show that the sample flow is focused near the center of the channel along the height direction. Due to the differing light absorption characteristics of the sample flow and the sheath fluid, the gray scale curves exhibit characteristic peaks in the central region, where the peak width corresponds to the focusing height. The experimental focusing heights are presented in Figure 11b. By calculating the average focusing height across the four analysis lines, the focusing heights under different vertical sheath-to-sample flow rate ratios were 16.70 μm ($R_{v2}=6$), 15.44 μm ($R_{v2}=8$), 13.66 μm ($R_{v2}=10$). Notably, the focusing height at $R_{v2}=8$ is consistent with the fluorescence focusing height measured in Section 3.2, indicating that both sample types achieve excellent vertical focusing within the PDMS chip. The focusing height is approximately 1.5 times the diameter of the ternary precursor particles, effectively constraining the particles within the microscope’s depth of field to ensure the acquisition of clear particle images from the top-view perspective.

**Figure 12:**
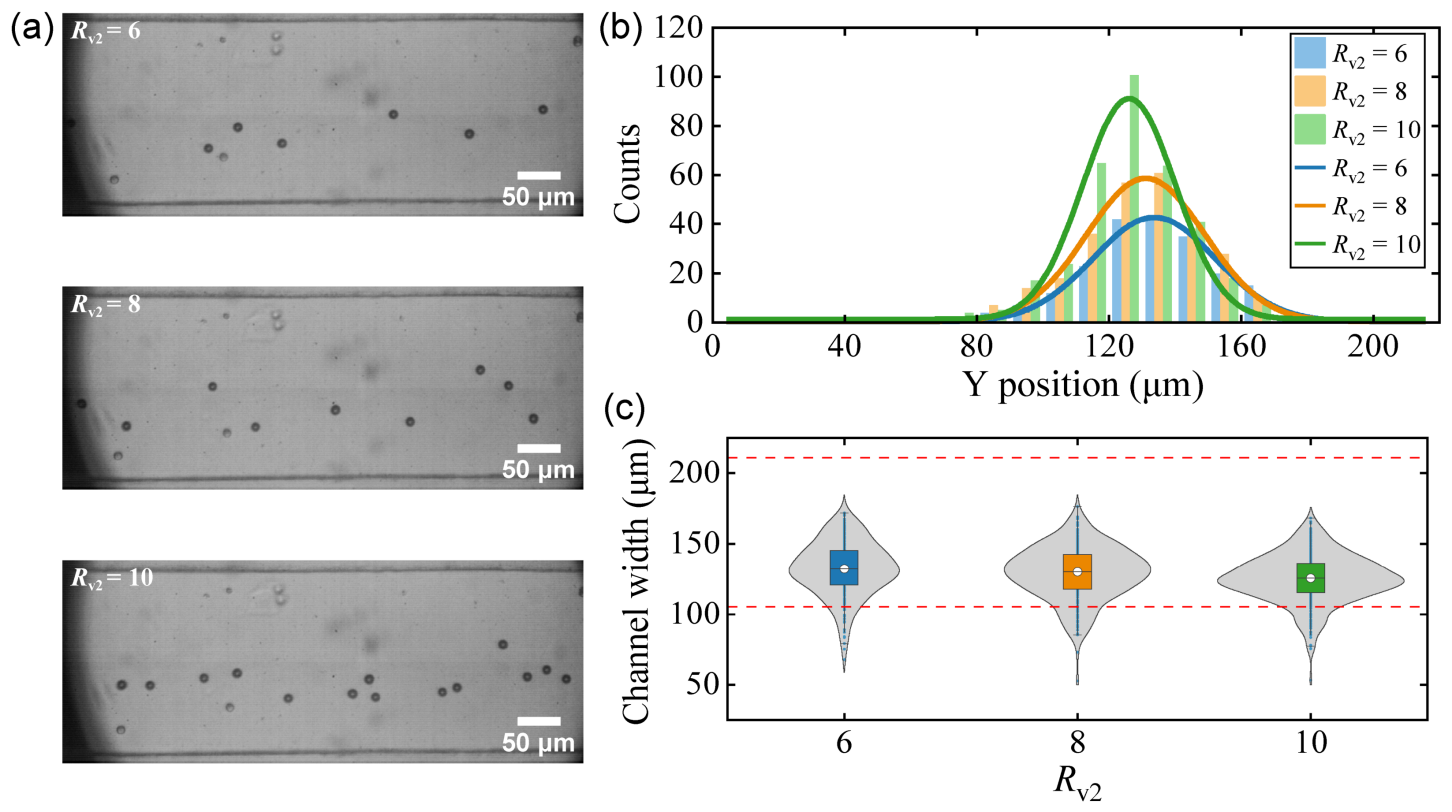
Analysis of horizontal particle focusing performance under different vertical focusing sheath flow rates. (**a**) Top-view precursor particle images downstream of junction J2 under different flow rate ratios $R_{v2}$. (**b**) Frequency distribution of particle Y positions. (**c**) Statistical distribution of particle Y positions.

Top-view images of the particles (xy-plane) were collected at various flow rate ratios using a 50× objective lens. The camera frame rate was set to 20,000 fps with an exposure time of 8 μs. The imaging region was located in the main channel downstream of the vertical sheath fluid confluence, as illustrated in Figure 12a, where particles are observed to be distributed near the center of the microchannel. For each flow rate ratio, 200 microscopic images were selected to analyze the spatial distribution of the particles. The statistical results, presented in Figure 12b, including histograms and fitting curves, demonstrate that the particle distribution in the y-direction is concentrated near the center of the channel width. Figure 12c provides the statistical distribution of particle positions across different flow rate ratios. At $R_{v2}=6$, a significant number of outliers are observed, suggesting that the sheath fluid pressure is insufficient to fully constrain the lateral displacement of the particles. The fewest outliers occur at $R_{v2}=8$, indicating the most stable flow state. When $R_{v2}=10$, the position distribution is centered near 125 μm, and the interquartile range (IQR), represented by the height of the boxes in the figure, is significantly reduced, signifying a more concentrated distribution range. A slight offset between the center of the particle distribution and the geometric center of the channel was noted. This discrepancy is attributed to fabrication tolerances in the PDMS chip, where the actual channel width is 211 μm, as well as alignment errors between different PDMS layers during the bonding process.

Furthermore, a statistical analysis was conducted on the particle microscopic images collected in 0.5 s during the experiment. The calculated particle throughput is summarized in Table 5. Under the parameter conditions of $Q_{s}=6\;\mu L/\min$, and $R_{v2}=10$ (equivalent to $Q_{\mathrm{sh}2}=60\;\mu L/\min$), the particle detection throughput reaches $4948\;s^{-1}$. This ribbon-like focused stream, characterized by its thin vertical profile and wide horizontal span, simultaneously ensures imaging accuracy with high vertical compression and maximizes throughput with a large lateral width to enable particle transport in multiple columns through the microchannel.

**Figure 13:**
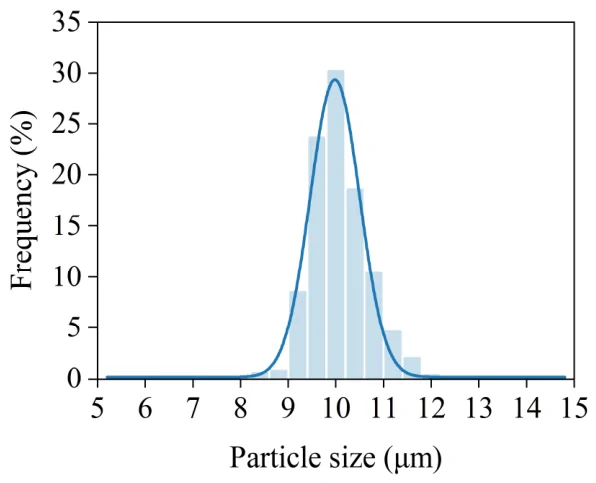
Particle size distribution of precursor particles.

**Figure 14:**
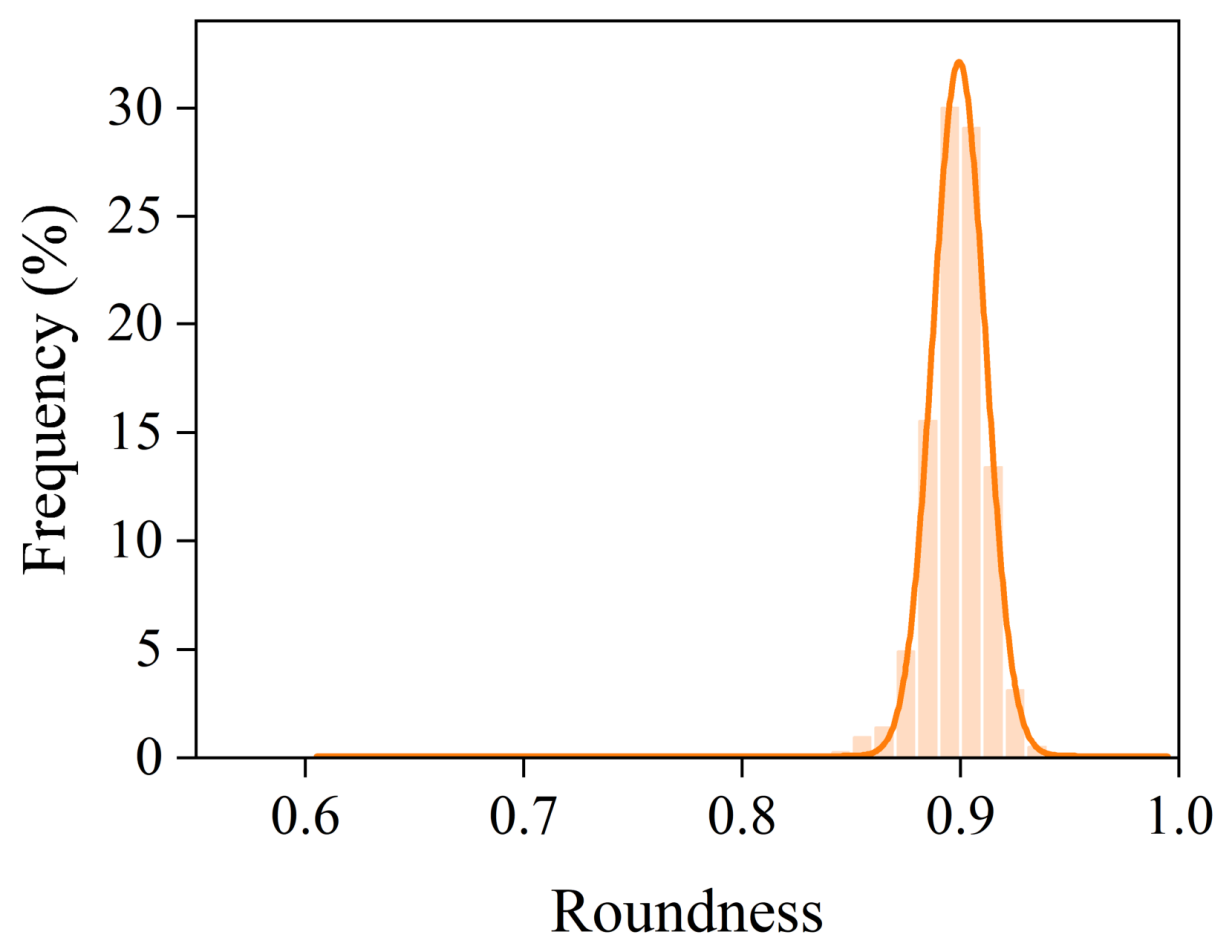
Roundness distribution of precursor particles.

Statistical analyses were also conducted on the microscopic characteristics of the precursor particles. Figure 13 shows the particle size distribution of the precursors, and Figure 14 shows the corresponding roundness distribution. The median particle size (D50) is 9.998 μm, which closely matches the measurements obtained via a commercial particle size analyzer. The circularity distribution is centered around 0.9 and exhibits a concentrated range, demonstrating that the precursor samples utilized in the experiments are near-spherical with relatively high uniformity.

## 4. Conclusions

In this study, we present a multi-layer PDMS chip in which a laser-etched middle layer is bonded with top and bottom layers fabricated via soft lithography. This design achieves three-dimensional (3D) hydrodynamic focusing through step-by-step horizontal and vertical compression of the sample. By minimizing the overlapping regions between channel layers, this structure reduces the technical difficulty of multi-layer PDMS bonding. Furthermore, the step-by-step focusing mechanism ensures stable sample transport within the microchannel, resulting in a flattened monolayer stream. The experimental results demonstrate that at a vertical sheath-to-sample flow rate ratio of $R_{v2}=8$, the microchannel compresses a sodium fluorescein solution to a vertical height of 15.69 μm. Under the same flow rate ratio, a ternary precursor solution is compressed to a height of 15.44 μm, with analysis of particle images collected in 0.5 s revealing a throughput of 2800 s^−1^. Additionally, it is confirmed that the dimensions of the 3D focused stream are primarily governed by the flow rate ratio. By setting flow rate ratio $R_{v2}=10$, the particle throughput reaches 4948 s^−1^, indicating that the microchannel allows for flexible flow adjustment to adapt high-throughput precursor particle imaging.

Nevertheless, two limitations of the current method should be noted. A relatively large distance between particles as they flow across the detection region limits the detection speed. Moreover, long-time continuous work leads to particle adhesion on microchannel surfaces, which requires periodic flushing. Future work will explore microchannel surface modification strategies to reduce particle adhesion. These findings provide critical technical support for the development of monitoring platforms for precursor microscopic characterization.

## Figures and Tables

**Table 1:** Results of mesh independence study.

Parameter *a*	Number of Mesh Elements	Cross-Section 1 Maximum Velocity (mm/s)	Cross-Section 2 Maximum Velocity (mm/s)	Cross-Section 3 Maximum Velocity (mm/s)	Cross-Section 4 Maximum Velocity (mm/s)	Mean Relative Error (%)
1	288,639	126.59	128.47	126.19	125.05	Baseline
0.9	367,679	129.42	126.95	126.90	126.89	1.36
0.8	490,191	128.76	129.94	130.48	130.19	2.59
0.7	721,561	132.04	128.42	131.61	126.88	2.53

**Table 2:** Simulation settings for structural parameter analysis.

Parameter Name	Parameter Value
Channel A Height Ratio $R_{\mathrm{ha}}$	1.5, 2, 2.5, 3, 3.5, 4
Channel B Height Ratio $R_{h}$	1, 2, 3, 4
Channel B Width Ratio $R_{w}$	0.25, 0.5, 0.75, 1
Channel Angle α (deg)	20, 30, 40, 50
Sample Flow Rate $Q_{\mathrm{sa}}$ (μL/min)	4
Flow Rate Ratio $R_{\mathrm{va}}$	10
Sample Flow Rate $Q_{s}$ (μL/min)	6
Flow Rate Ratio $R_{v1}$	0.5
Flow Rate Ratio $R_{v2}$	6

**Table 3:** Simulation settings for flow rate parameter analysis.

Parameter Name	Parameter Value
Sample Flow Rate $Q_{\mathrm{sa}}$ (μL/min)	4
Flow Rate Ratio $R_{\mathrm{va}}$	8, 10, 12, 14, 16, 18
Sample Flow Rate $Q_{s}$ (μL/min)	6
Flow Rate Ratio $R_{v1}$	0.25, 0.5, 0.75, 1, 1.25
Flow Rate Ratio $R_{v2}$	2, 4, 6, 8, 10

**Table 4:** Focused stream dimensions under different absolute flow rates.

Description	Mean (μm)	Standard Deviation (μm)
Simulated focused stream width	92.3	0.29
Experimental focused stream width	65.97	1.09
Simulated focused stream height	12.5	0
Experimental focused stream height	15.97	0.42

**Table 5:** Particle detection throughput at different flow rate ratios $R_{v2}$.

Flow Rate Ratio $R_{v2}$	6	8	10
Number of particles in 0.5 s	1059	1400	2474
Particle detection throughput (particles/s)	2118	2800	4948

**Figure A1:**
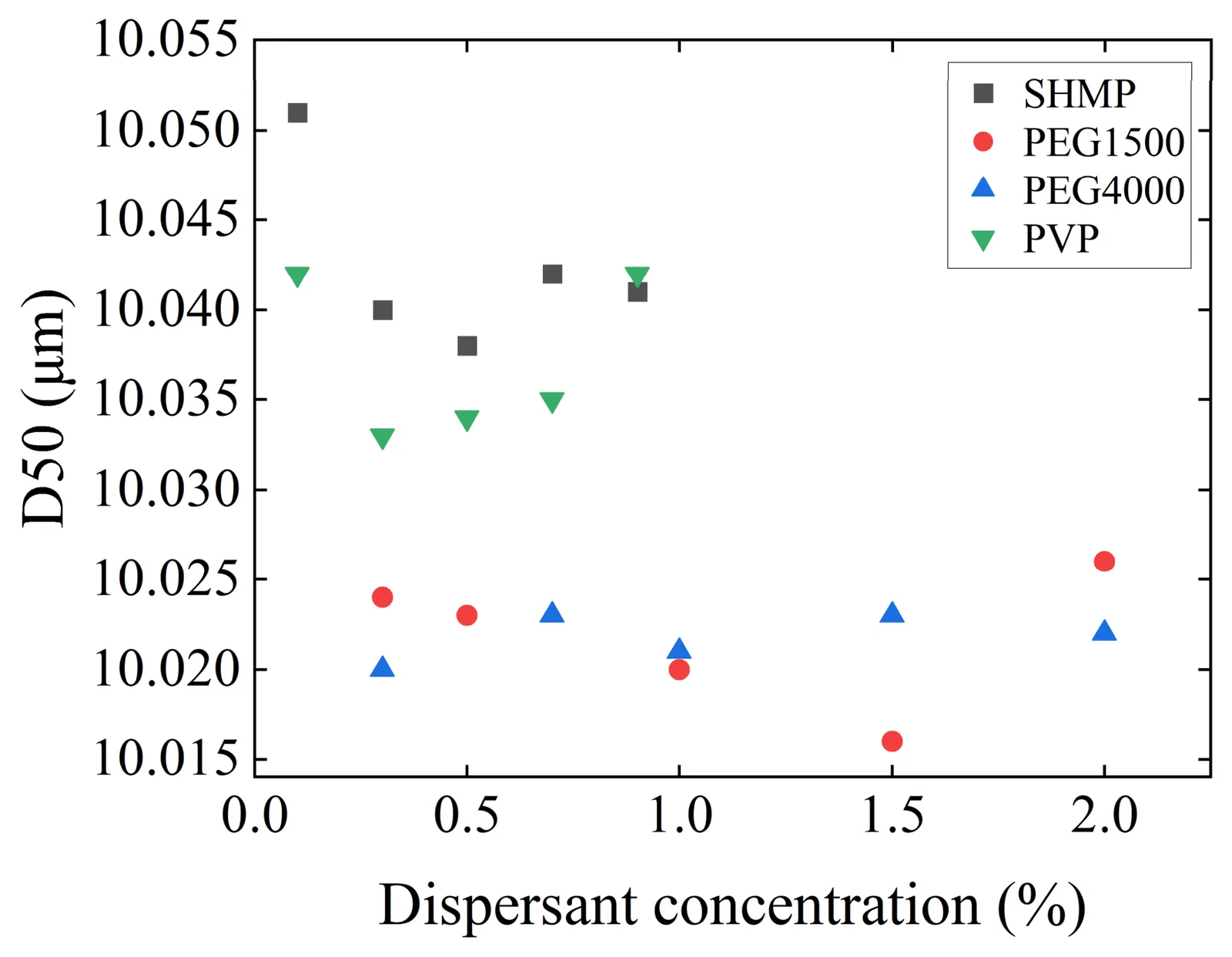
Median particle size (D50) of precursor particles with the addition of different dispersants.

**Figure A2:**
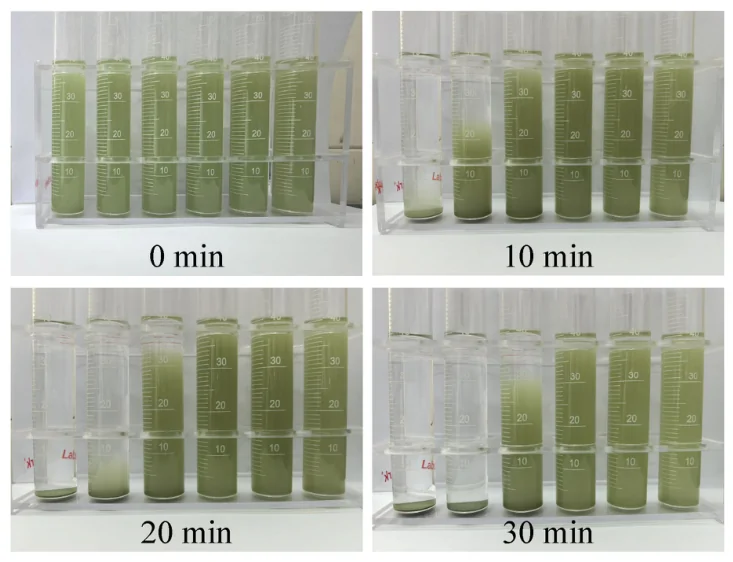
Precursor suspensions containing sodium carboxymethyl cellulose (CMC) at concentrations of (from left to right) 0%, 0.2%, 0.4%, 0.6%, 0.8%, 1%.

## Data Availability

The data presented in this study are available on request from the authors.

## Appendix A. Pretreatment of Ternary Precursor Suspension

Initial stability analysis was performed using Zeta potential measurements (Litesizer 500, Anton Paar GmbH, Graz, Austria), which yielded a mean value of −1.849 mV across three trials. This indicates that while the particles carry a slight negative charge, the electrostatic repulsion between them is insufficient to prevent proximity and subsequent aggregation, leaving the particle system in an unstable state. Furthermore, the measured viscosity of the solution was 1.84 mPa·s (MCR 92, Anton Paar GmbH, Graz, Austria). According to Stokes’ Law, the sedimentation velocity of a particle is inversely proportional to the fluid viscosity. Therefore, the low viscosity of the ternary precursor solution leads to rapid particle settling. Consequently, efforts to improve the stability of the particle system focus on two primary objectives: suppressing aggregation and hindering sedimentation. This is achieved by increasing electrostatic repulsion to overcome van der Waals forces, thereby reducing particle clustering, and by modifying the rheological properties of the solution to effectively retard the sedimentation process.

To achieve the aforementioned objectives, dispersants and thickeners were selected for the pretreatment of the ternary precursor solution. The stabilization mechanism of the dispersants involves their adsorption onto the particle surfaces, providing robust electrostatic repulsion or steric hindrance. The thickeners function by forming a weak yet continuous three-dimensional network structure through molecular chain entanglement, hydrogen bonding, and van der Waals interactions. This structure increases the zero-shear viscosity of the suspension and may generate a specific yield stress to counteract the gravitational force of the precursor particles, thereby effectively inhibiting sedimentation. Considering the distinct mechanisms associated with different additives, several types of dispersants were selected for evaluation, specifically sodium hexametaphosphate (SHMP, Xilong Scientific Co., Ltd., Shantou, China), polyethylene glycol (PEG, Xilong Scientific Co., Ltd., Shantou, China), polyvinylpyrrolidone (PVP, Xilong Scientific Co., Ltd., Shantou, China), and sodium dodecyl sulfate (SDS, Sinopharm Chemical Reagent Co., Ltd., Shanghai, China), with sodium carboxymethyl cellulose (CMC, Macklin Biochemical Co., Ltd., Shanghai, China) serving as the thickener. Experiments were conducted to investigate the effects of various dispersants and thickeners on the Zeta potential, particle size distribution, sedimentation rate, and rheological profiles of the precursor solution.

Ultrasonication was performed using a SCF-105B sampling system (OMEC Instruments Co., Ltd., Zhuhai, China), with the obscuration maintained within a specific range. The process parameters were set to an ultrasonication time of 30 s and an intensity of 100% to investigate the influence of different types and concentrations of dispersants on the particle size distribution. The dispersant concentrations and the corresponding measured median particle sizes of the precursors are illustrated in Figure A1. Based on the median particle size (10.036 μm) measured in the absence of any additives, it was observed that the selected dispersants had a negligible effect on the median particle size of the precursors.

The sedimentation method was employed to evaluate the influence of varying concentrations of sodium carboxymethyl cellulose (CMC) on the suspension stability of the ternary precursor particles. CMC solutions were prepared at concentrations of 0.2%, 0.4%, 0.6%, 0.8%, and 1.0% (40 mL each), with 40 mL of deionized water serving as the control group. Subsequently, 1 mL of the ternary precursor slurry was added to each solution. These mixtures were transferred to graduated colorimetric tubes, shaken thoroughly, and then stood undisturbed prior to analysis. Figure A2 illustrates photographs of the precursor suspensions containing different CMC concentrations at t = 0, 10, 20, and 30 min. These photographs indicate that after 10 min of standing, significant particle sedimentation occurred in the suspensions with 0% and 0.2% CMC. After 20 min, minor sedimentation was observed in the 0.4% CMC suspension. By the 30 min mark, the suspensions containing 0.6%, 0.8%, and 1.0% CMC exhibited only negligible particle sedimentation.

To ensure the suspension stability of the precursor particles during detection, CMC concentrations of 0.6%, 0.8%, and 1.0% were considered viable options. Given that high-viscosity solutions necessitate elevated flow rates for stable transport within microchannels, the 0.6% CMC solution was ultimately selected as the thickener. This concentration was chosen to enhance the suspension stability of the particles while maintaining compatible fluidic delivery conditions within the microfluidic chip.

Zeta potential measurements were conducted on the precursor samples treated with the 0.6% CMC solution, yielding a mean value of −30 mV across three trials. The measured viscosity was 15 mPa·s and the density was approximately 1300 kg/m^3^. These results indicate that the precursor particles can maintain a stable suspension over a sufficient duration to meet the requirements for detection in a microfluidic chip.
